# Life satisfaction prevents decline in working memory, spatial cognition, and processing speed: Latent change score analyses across 23 years

**DOI:** 10.1192/j.eurpsy.2022.19

**Published:** 2022-04-19

**Authors:** Nur Hani Zainal, Michelle G. Newman

**Affiliations:** 1 Massachusetts General Hospital, Harvard Medical School, Boston, Massachusetts, USA; 2 Department of Psychology, The Pennsylvania State University, University Park, Pennsylvania, USA; 3 National University of Singapore, Singapore

**Keywords:** Latent change, life satisfaction, processing speed, spatial cognition, working memory

## Abstract

**Background:**

Within-person growth in life satisfaction (LS) can protect against declines in cognitive functioning, and, conversely, over time. However, most studies have been cross-sectional, thereby precluding causal inferences. Thus, we used *bivariate dual latent change score modeling* to test *within-person* change-to-future change relations between LS and cognition.

**Method:**

Community adults completed in-person tests of verbal working memory (WM), processing speed, spatial cognition, and an LS self-report. Five waves of assessment occurred across 23 years.

**Results:**

Reduction in LS predicted future decreases in spatial cognition, processing speed, and verbal WM (|*d*| = 0.150–0.354). Additionally, depletion in processing speed and verbal WM predicted a future decrease in LS (*d* = 0.142–0.269). However, change in spatial cognition did not predict change in LS (|*d*| = 0.085).

**Discussion:**

LS and verbal WM and processing speed predicted one another across long durations. Evidence-based therapies can be augmented to target LS and cognition.

The challenges and opportunities of our daily lives require most of us to harness specific cognitive function abilities for successful navigation. These cognitive capacities include verbal working memory (WM; fluency to process mentally and change auditory data in real-time) [1], spatial cognition (ability to cognitively generate, sustain, retrieve, and alter visual objects) [2], and processing speed (capacity to fulfill rudimentary or intricate tasks that require persistent attention efficiently) [3]. These cognitive indices have been reliably correlated with various markers of biopsychosocial health. Diverse health indicators include mood regulation [4,5], physical health [6,7], school performance [8], work productivity [9], and quality of personal and professional relationships [10]. This is likely because spatial cognition, verbal WM, and processing speed are related to myriad basic attentional and complex higher-order executive functioning skills [11] and emotion modulation tactics [12]. Thus, enhancing our understanding of the predictors and outcomes of these aspects of cognitive function is essential.

It has been theorized that factors which facilitate the growth of psychological well-being, such as life satisfaction (LS), may prevent future long-term decline in cognitive abilities [13,14]. LS is the subjective global appraisal of the degree to which cumulative experiences thus far have been, on balance, fulfilling, and rewarding in the realms of health, relationships, career, and lived environments [15]. Relatedly, empirical evidence has shown that LS is a construct distinct from dispositional negative affect or worry (tendency to assume the worst) and depression (prolonged low mood) at a single time point and across long developmental periods [16,17].

Reduction in LS may lead to subsequent cognitive function deterioration across long durations via suboptimal lifestyle choices such as infrequent exercise and unhealthy diet and nutrition [18,19]. Failure to engage in health-promoting activities could lead to increased inflammation in executive functioning and related brain regions and weakened cardiovascular strength, thus causing long-term adverse effects of aging on cognitive function [20–23]. Such ideas are consistent with *scar theories* which argue that behavioral inactivity and repetitive negative thinking that may accompany a reduction in LS could over time negatively impact cognitive function abilities [24,25]. This process may occur through increased allostatic load (i.e., stress-linked wear and tear of the hypothalamic-pituitary-axis and cognitive function-related brain regions) [26] across long periods. Thus, a decrease in LS may dovetail with worsening of spatial cognition, verbal WM, and processing speed in the long term.

As yet, six longitudinal studies have tested the aforesaid idea that reduced LS and related concepts would be associated with subsequent cognitive function decline. For example, among older Swedish community adults, diminished LS coincided with a decrease in self-rated cognitive capacity from working life to post-retirement [27]; however, whether LS would forecast *performance-based* cognitive function scores could not be deduced from that study. Other studies suggest such a possibility. Using latent growth curve modeling, Wilson et al. [28] and Salthouse [29] observed that *between persons*, lower scores on LS and related indices (e.g., satisfaction with relationships) predicted a more rapid decline in processing speed, global cognition, and memory over 6–13 years in early, middle, and late adulthood. Likewise, using hierarchical linear modeling, *within persons*, an 8-year decrease in psychological well-being was correlated with reductions in processing speed, memory, and global cognition among English midlife and older community adults during that period [30]; despite that, lead–lag relations were not examined in the study. A Switzerland-based study [31] that tested lead–lag associations showed that reduced LS predicted a 6-year decline in set-shifting (i.e., the adeptness to change from one mental mode to another [32]); however, due to its two-wave design, *change*-to-future *change* relations could not be inferred. Relatedly, within persons, a lower prior level of LS resulted in a greater 1.5–2-year decline in perceptual speed among older German adults across six waves of assessment [33].

Simultaneously, theorists have proposed that factors impeding goal attainment, such as a decline in cognitive function, may predict a reduction in LS across long durations [34,35]. This is because such executive functioning-related capacities are essential to engender and sustain progress and a sense of fulfillment in school, career, relationships, and other realms reflective of biopsychosocial health across time [36,37]. It is also plausible that increased cognitive function, such as processing speed, spatial cognition, and verbal WM, are protective against depletion of LS across development as these resources aid optimal problem definition, planning, and skillful decision-making [38,39]. Such cognitive resources-related strategic approaches include sacrificing short-term relief or pleasure to fulfill long-term aspirations [40]. Overall, it is tenable that a decline in verbal WM, processing speed and spatial cognition would predict a subsequent decrease in LS.

Thus far, seven studies have tested the proposition that a decline in LS would be related to future cognitive function deficits or vice versa. One earlier study evidenced no relation between LS and global cognition 6 years later in middle- to older-aged adults [41]. Conversely, middle-aged and older men who displayed stronger verbal WM and processing speed reported higher LS or positive affect following 7–12 years [42,43]. Similarly, another study that used ordinary least squares (OLS) regression demonstrated that higher baseline spatial cognition or processing speed scores predicted greater LS after 3 years in younger, middle-aged, and older adults [44]. Moreover, stronger WM ability predicted higher trait and daily levels of LS among college students [45]. In addition to performance-based cognitive function measures, two recent studies [27,46] showed that a decline in subjective cognitive capacity was associated with a later reduction in LS among adults transitioning to retirement.

Despite the progress made thus far to understand the relations between LS and cognitive function, literature still presents with several limitations the current study aimed to remedy. First, most studies testing LS–cognitive function relations were cross-sectional (e.g., [47–49]), thus precluding causal inferences due to the absence of temporal precedence. Second, most prior studies on this topic used OLS regression, latent growth modeling, and cross-lagged panel models, which permit conclusions on *between*-person, but not *within-*person, associations [50,51]. This is problematic as theories above on LS–cognitive function connections imply how *within-person* changes in each unique variable predict one another across the lifespan. Furthermore, relations between- and within-persons can differ in magnitude and direction [5,52], underlining the need to tether *within-person* analytic methods to test preceding theories. Therefore, the present study used *bivariate dual latent change score* (BLCS) modeling. BLCS modeling is an advanced longitudinal structural equation modeling (SEM) technique that could examine *within-person* change-to-future change relations among unique variables [53,54]. BLCS reduces biases arising from between-person effects, measurement error, autoregressive self-feedback loops, baseline and prior scores, and regression to the mean [55]. BLCS thus allows a better understanding of how a change in cognitive function at a time lag is linked to a change in LS at a future time lag (and conversely) [16].

A better comprehension of the *within-person* factors that predict the decline in LS and cognitive function is imperative. Globally, societies are currently grappling with increasing economic, psychosocial, and public health burdens linked to diverse mental illnesses (e.g., depression and anxiety) and neurocognitive disorders (e.g., dementia) that compromise LS [56–58]. Studies that identify the risk factors for decline in cognitive function and LS may be helpful for developing effective prevention, early identification, and treatment strategies. Moreover, *within-person change* lies at the heart of clinical science [52,59]. Thus, based on the preceding theories and data, we used BLCS modeling to test lead–lag associations between LS and cognitive function across five time points spanning 23 years in a community adult sample. First, we hypothesized that within persons, a decline in LS at a prior time lag would be significantly related to decreased verbal WM, processing speed, and spatial cognition at the next adjacent time lag (Hypothesis 1). Likewise, we predicted that within-person decline in processing speed, verbal WM, and spatial cognition at a previous time lag would be substantially associated with a reduction in LS at the adjacent time lag (Hypothesis 2).

## Method

### Participants

The current study examined participants (*n* = 560) who gave permission to undergo face-to-face neuropsychology testing and tocomplete in-person self-report measures such as the LS questionnaire. Forty participants were removed from our analyses as they were diagnosed with dementia, leaving a final sample of 520 participants.^1^ Of the 520 participants, 54.8% identified as probable or definite fraternal twins, 30.8% as identical twins, and the remaining 14.4% mentioned they did not know the nature of their twinship. In addition, 52% were reared together, and the remaining 48% were reared apart. The current sample were middle- to older-aged adults at baseline (*M* age = 59.61 years, *SD* = 8.98, range = 40.57–84.11; refer to Table S1 in the Supplementary Material [SM] for demographics at each time point). Females comprised 58% (*n* = 302) of the sample. Concerning formal education, 8.46% received elementary to lower secondary school, 21.20% had a high school education, and 70.40% attended college or higher academia, which included courses to acquire specialized skill sets (e.g., forestry and business).

### Procedures

Using the Swedish Adoption/Twin Study of Aging publicly available dataset, the present study conducted a theoretically informed secondary analysis. The Karolinska Institutet provided ethical approval, and all participants gave voluntary informed consent. We were interested in the relations between LS and cognitive function (processing speed, verbal WM, and spatial cognition scores) instead of genetic heritability [62]. The study comprised five waves of assessment during these years: 1987 (Time 1; T1), 1990 (Time 2; T2), 1993 (Time 3; T3), 2004 (Time 4; T4), and 2007 (Time 5; T5). Due to funding deficits, a sizeable 11-year gap occurred between 1993 and 2004, and in-person cognitive testing was unavailable during this period. This led to five available time points with four sequential time lags ranging from 3 to 11 years. Thus, the present study examined if within-person change at a time lag (Δ*T*) in a variable was associated with change at the next sequential time lag (Δ*T* + 1) in another variable. The following measures were administered at all five time points.

### Measures

#### Life satisfaction

An 8-item self-report measure of LS adapted from [63]) was used. Respondents endorsed items on a 5-point Likert scale, ranging from 1 = *strongly disagree* to 5 = *strongly agree.* It showed satisfactory-to-good between-person reliability (Cronbach’s *α* = .70–.79) and within-person reliability (*α* = .70) in the current study. Furthermore, the measure demonstrated good retest reliability (*r* = .79) [63], as well as strong convergent and discriminant validity [64,65]. Table S2 in the SM lists the items in this measure and details how the eight items in the current scale were derived from the original 13-item scale [64].

#### Spatial cognition

The Koh’s block design (BD) and card rotations (CR) tests assessed spatial cognition. Koh’s BD test parallels the Wechsler’s Adult Intelligence Scale [66] BD subtest; participants were instructed to replicate seven increasingly difficult square- and triangle-filled figures with two-colored blocks. In addition, the CR test required participants to correctly select, upon mental rotation, one out of four items that matched a target figure on display. Scores for both tests indicated the total completion time (in seconds) for each presented figure. These assessments have shown strong between-person reliability (*α* = .81–.85) and within-person reliability (*α* = .90) herein, high 33-day test–retest reliability (*r* = .90) [67], and good discriminant and convergent validity [68].

#### Processing speed

The widely used oral version of the symbol digit modalities (SDM) test [69,70] measured processing speed capacity.^2^ Participants orally indicated each integer (ranging from 1 to 9) linked to a specific unique symbol. The SDM test was administered in two blocks of 50 integers, each for 45 s. Possible scores ranged from 0 to 100. In the current study, the SDM test showed strong between-person (*α* = .90–.92) and within-person reliability (*α* = .90). Furthermore, it has shown strong 2-week retest reliability (*r* = .74), convergent and discriminant validity, with scores of measures of different constructs [71–73].

#### Verbal WM

The digit span (DS) test-backward was administered to assess verbal WM by instructing participants to register and recite three-to-nine number sequences of growing length in the reverse direction. The DS test-backward score was computed by summing the total number of digit sequences accurately recalled. Although DS test-forward measures verbal WM repetition, DS test-backward captures WM and attentional control processes reliably. It has excellent between-person (*α* = .93) and within-person reliability (*α* = .89), as well as retest reliability (*α* = .83) [74]. Moreover, it has strong discriminant and convergent validity [75,76].

### Data analyses

All preprocessing and data analyses were conducted with *R* version 4.1.0 [77] and *RStudio* Version 1.4.1717. First, using the *psych R* package [78], we inspected the data, determined that all variables were normally distributed, and observed no outliers (see Table S3 in the SM for descriptive statistics and correlation matrix of study variables). The *psych R* package [78] was also used to conduct psychometric analyses (e.g., compute reliabilities or internal consistency of scores on measures). Next, a series of SEM analyses were performed. To evaluate SEM model fit, these indices were used: Chi-square with degrees of freedom (χ^2^(*df*)) and related *p*-value; confirmatory fit index (CFI) [79]; and root-mean-square error of approximation (RMSEA) [80]. Moreover, nesting within twins was accounted for by utilizing the *lavaan.survey R* package [81]. Furthermore, maximum likelihood with robust sandwich estimators were used.

Figure S1 in the SM details the number of participants who completed the in-person cognitive function tests and the LS self-report at each time point. Missingness (a total of 24% of all observations) was managed by using multiple imputation with the raw item scores following recommended guidelines [82]. Data were aggregated across 100 multiply imputed datasets with the predictive mean matching algorithm, each with a maximum of 100 iterations; a gold standard approach in aging research [83]. Auxiliary variables that could have been related to dropout status (age, gender, education, baseline scores of cognitive functioning, and LS) were included in the multiple imputation models at all time points; a method that adjusts for any systematic trends in dropouts and yields parameter estimates aligned with the missing at random assumption. Moreover, multiple imputation (vs. complete case analysis) tends to produce unbiased, efficient, and precise parameter and standard error estimates that mirror those derived from complete datasets, even if total missingness exceeds 50% [84,85].^3^

As a precursor to longitudinal SEM, we tested for longitudinal measurement invariance of all the measures to determine if their psychometric properties were reproducible across all time points [86]. All levels of invariance were gradually tested in this order with increasingly restrictive models: *configural* (equal factor structure, varying factor loadings [λs], item intercepts [τs], and item residual variances [θs]); *metric* (equal factor structure, λs, varying τs, and θs); *scalar* (equal factor structure, λs, and τs, varying θs); and *strict* (equal factor structure, λs, τs, and θs). Measurement invariance was indicated by trivial changes in the practical fit indices; ΔCFI < −.010 or ΔRMSEA < +.030 when comparing the more (vs. less) constrained models [87,88].

Next, we conducted a series of LCS models to test Hypotheses 1 and 2. Latent change scores were computed by fixing the regression path between scores at a prior time point and the next adjacent time point equal to 1 [50]. This indicated that a certain amount of the subsequent score was equivalent to the prior score, and the residual score was construed as the latent change score. Equations (1) and (2) below denote univariate dual LCS models for LS and cognitive function variables. ∆*C* and ∆*L* signify the latent change in each cognitive function variable and LS at a time lag (∆*T*), respectively. The term *α* indicates the between-person constant change parameter of the slope of a variable, and *β* relays *within-person* proportional effects (autoregressive self-feedback loops of change in a variable forecasting future change in itself).
(1)

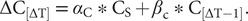



(2)

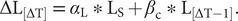

BLCS models were then conducted to test dynamic relations between LS and cognitive function within and between persons. By combining attributes of the cross-lagged panel and latent growth curve models, BLCS models examine change in each variable from one time point to an adjacent time point in a time-sequential manner across all time points [89]. It computes *within-person coupling effects* (*δ*; cross-lagged effects of change in a variable at a time lag predicting change in another variable at a successive time lag). Simultaneously, BLCS models minimize biases stemming from measurement unreliability, regression to the mean, baseline scores and between-persons (or trait-level) intercepts, variances, constant change, and other model parameters [90]. Equations (3) and (4) denote bivariate LCS models examined herein:
(3)

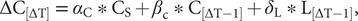



(4)

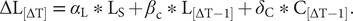

**Figure 1.**
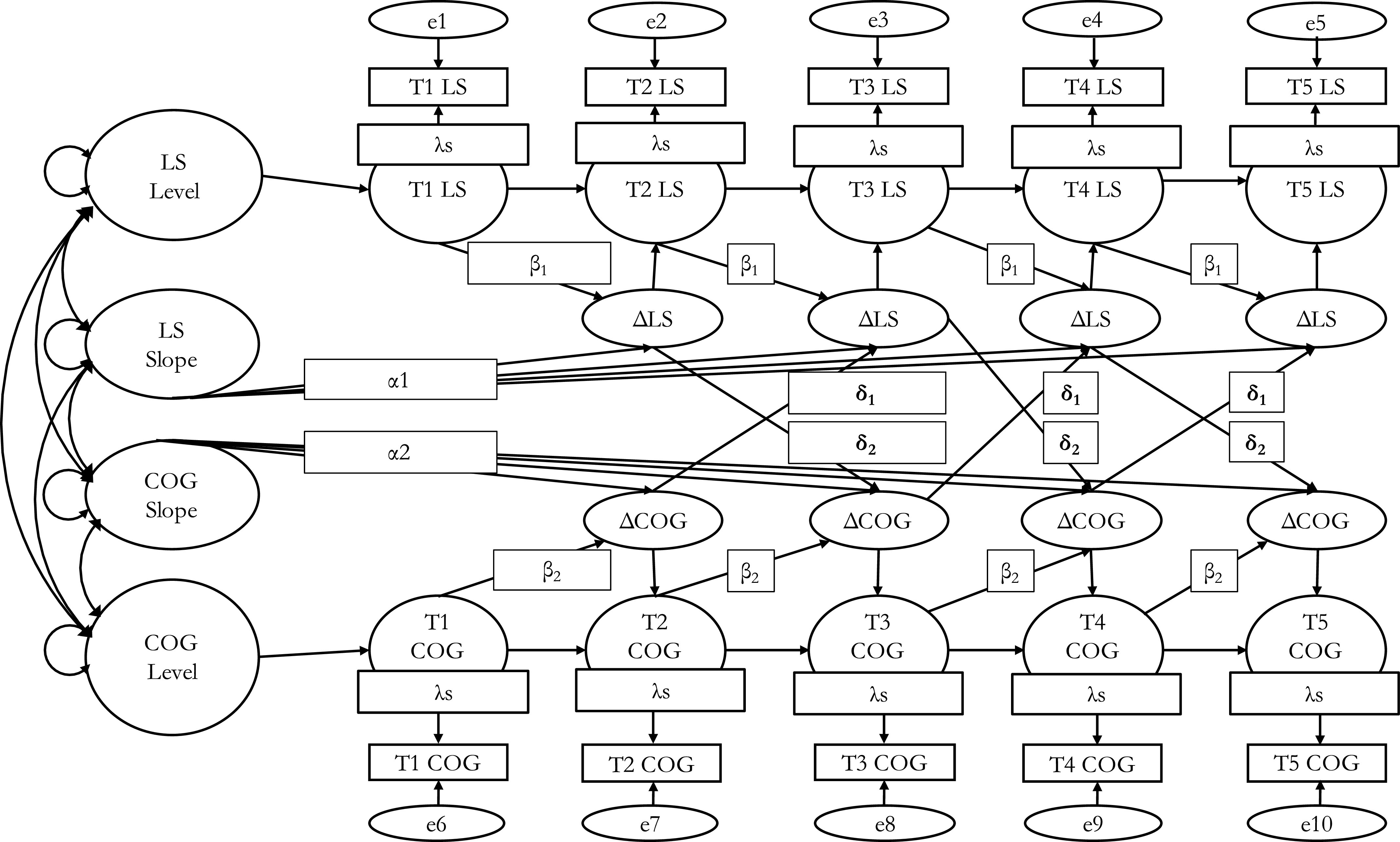
Bivariate dual latent change score model of LS and cognitive function. *Abbreviations:* COG, global cognition; e, item-level residual error; LS, life satisfaction; T1, Time 1 (1987); T2, Time 2 (1990); T3, Time 3 (1993); T4, Time 4 (2004); T5, Time 5 (2007); *α*, between-person constant change; *β*, within-person proportional change (change in a variable predicting future change in itself); Δ, change in a variable; *δ*, within-person cross-domain coupling effect (change in a variable predicting future change in another variable); *λ*, factor loading.

Furthermore, permitting within-person change-to-future change cross-construct coupling effects to be freely estimated across time-lags increases standard errors. Thus, following recommendations [91], we fixed the cross-construct coupling effects to be equal. Figure 1 displays the graphical representation of a BLCS model and embedded parameters. Furthermore, to account for differences in time-lag length herein (i.e., 3 vs. 11 year), we conducted a chi-square (∆*χ*^2^(*df*)) difference test [92] to examine if time-lag length significantly moderated critical parameter estimates (i.e., cross-construct coupling effects). Moreover, to determine if a change in LS predicting future change in cognitive functioning was stronger than vice versa, we conducted a ∆*χ*^2^(*df*) test of BLCS models with and without equality constraints on the within-person coupling effects. In addition, given multiple comparisons of parameter estimates within and across BLCS models, we used the mixture distribution approach of alpha correction recommended by Ke and Wang [93] and Wang and Yang [94], which reduces the odds of Type I error in BLCS models. Based on simulations using this approach, these authors recommend that for our current sample size (*N* = 520) and our five measurement points, a value of *p* ≤ 0.029 would be the optimal cutoff to reduce the likelihood of Type I error [93,94]. Furthermore, Cohen’s *d* effect sizes were computed with the formula *d* = *β*/(*SE*(*β*)) × √(2/*n*) [95], such that *d* values of 0.2, 0.5, and 0.8 indicated small, moderate, and large effects, respectively.

### Power analysis

A Monte Carlo power analysis mirroring study conditions (a gold standard recommended approach) was conducted using the *RAMpath R* package [96]. To detect within-person change-to-future change cross-construct (coupling effects) and related parameter estimates (e.g., mean and variance) with conservative and small effect sizes ranging from |*d*| = 0.10 to 0.20, the current study had 97.4–100.0% power. Thus, the current study had an adequate sample size to conduct BLCS analyses.

## Results

### Longitudinal measurement invariance

As shown in Table 1, across all time points, the two-factor CFA model that included LS and each cognitive function variable displayed measurement invariance at the *strict* level (equal λs, τs, and εs). This meant that measures herein had comparable psychometric properties and could be construed along with the same metric at all time points. Thus, longitudinal SEM was appropriate for this dataset.

**Table 1.** Longitudinal measurement invariance of the four-factor model of LS and cognition.

	WLSMV χ^2^	*df*	*p*	CFI	RMSEA
Four-factor model of LS and cognition (spatial WM, verbal WM, and processing speed)					
Time 1 (1986–1988)	97.564	50	<0.001	.971	.050
Time 2 (1989–1991)	104.132	50	<0.001	.967	.051
Time 3 (1992–1994)	109.914	50	<0.001	.961	.053
Time 5 (2005–2007)	84.893	50	<0.001	.963	.052
Time 6 (2007–2008)	84.443	50	<0.001	.955	.061
Level of measurement invariance					
Configural (varying *λ*, *τ*, *ε* across time)	489.792	254	<0.001	.964	.053
Metric (equal *λ*, varying *τ*, *ε* across time)	610.792	282	<0.001	.950	.059
Scalar (equal *λ*, *τ*, varying *ε* across time)	706.402	314	<0.001	.940	.061
Strict (equal *λ*, *τ*, *ε* across time)	793.346	354	<0.001	.933	.061
Tests of measurement invariance					
Configural invariance vs. metric invariance	Δχ^2^(*df* = 28) = 121.000, *p* < 0.001, ΔCFI = −.014, ΔRMSEA = .007				
Metric invariance vs. scalar invariance	Δχ^2^(*df* = 32) = 95.610, *p* < 0.001, ΔCFI = −.001, ΔRMSEA = .002				
Scalar invariance vs. strict invariance	Δχ^2^(*df* = 40) = 86.944, *p* < 0.001, ΔCFI = −.007, ΔRMSEA = −.000				

*Abbreviations:* CFI, confirmatory fit index; LS, life satisfaction; RMSEA, root-mean-square error of approximation; WM, working memory; εs, residual variances; λs, factor loadings; τs, intercepts.

### Univariate latent change score models

Table 2 demonstrates that the model fit indices for the ULCS models were good for LS (χ^2^(*df* = 13) = 50.479, *p* < 0.001, CFI = .963, RMSEA = .058), spatial cognition (χ^2^(*df* = 13) = 63.031, *p* < 0.001, CFI = .969, RMSEA = .086), verbal WM (χ^2^(*df* = 13) = 31.780, *p* < 0.001, CFI = .954, RMSEA = .053), and processing speed (χ^2^(*df* = 13) = 75.714, *p* < 0.001, CFI = .941, RMSEA = .097). Within persons, higher level of cognitive functioning at a prior time point was significantly associated with greater rise in cognitive functioning at the next time lag for all specific cognitive functioning indices (spatial cognition: *β* = 0.341, 95% CI [0.192, 0.491], *p* < 0.001, *d* = 0.278; verbal WM: *β* = 0.733, 95% CI [0.213, 1.253], *p* = 0.006, *d* = 0.172; processing speed: *β* = 0.234, 95% CI [0.114, 0.354], *p* < 0.001, *d* = 0.238). However, level of LS at a previous time point was not significantly related to future change in itself within persons (*β* = −0.043, 95% CI [−0.247, 0.161], *p* = 0.680, *d* = −0.036).

**Table 2.** Univariate latent change score models of each variable.

	LS			Spatial cognition		
Within-person proportional change (*β*)	*β*	[95% CI]	ES *d*	*β*	[95% CI]	ES *d*
	−0.043	[−0.247, 0.161]	−0.036	0.341^***^	[0.192, 0.491]	0.278
Initial status						
Mean	2.556^***^	[2.494, 2.618]	8.364	36.683^***^	[35.619, 37.748]	4.190
Variance	0.420^***^	[0.348, 0.493]	0.624	127.403^***^	[111.697, 143.108]	0.986
Constant change (*α*)						
Mean	0.156	[−0.371, 0.683]	0.038	−13.924^***^	[−19.089, −8.759]	−0.328
Variance	0.019^***^	[0.010, 0.139]	0.155	14.539^***^	[1.931, 27.147]	0.140
Correlation between initial status and *α*	−0.044	[−0.112, 0.024]	−0.007	−41.966^***^	[−62.096, −21.835]	−0.253
Residual error						
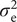	0.147^***^	[0.130, 0.165]	0.992	29.435^***^	[26.605, 32.265]	1.264
Model fit						
χ^2^	50.479			63.031		
*df*	13			13		
*p*	<.001			<.001		
CFI	.963			.969		
RMSEA	.058			.086		

*Abbreviations:* CFI, confirmatory fit index; *df*, degrees of freedom; LS, life satisfaction; RMSEA, root-mean-square error of approximation; VSP, visual–spatial processing.

* *p* < 0.05.

** *p* < 0.01.

*** *p* < 0.001.

### Bivariate dual latent change score models

#### Life satisfaction and spatial cognition

Table 3 shows that the BLCS model examining within-person change-to-future change associations between LS and spatial cognition had good model fit (χ^2^(*df* = 40) = 118.780, *p* < 0.001, CFI = .968, RMSEA = .063). Within persons, prior greater decrease in LS at a 3–10-year time lag was significantly linked to higher future reduction in spatial cognition ability at the following time lag (*β* = −12.661, 95% CI [−22.892, −2.430], *p* = 0.015, *d* = −0.150). However, change in spatial cognition at a time lag was not significantly related to change in LS at the next adjacent time lag (*β* = −0.030, 95% CI [−0.072, 0.013], *p* = 0.177, *d* = −0.085). In addition, the cross-lagged change-to-future change association was significantly stronger for LS predicting spatial cognition ability than the reverse direction (Δχ^2^(*df* = 1) = 4.167, *p* = 0.041). Furthermore, time-lag length did not significantly moderate cross-construct coupling effects (Δχ^2^(*df* = 1) = 2.323, *p* = 0.127). Moreover, time-lag length differences (3 vs. 11 year) did not significantly moderate the change-to-future change cross-construct coupling effects between spatial cognition and LS (∆*χ*^2^(*df* = 2) = 2.009, *p* = 0.366).

**Table 3.** Bivariate latent difference score models of spatial cognition and LS.

Parameter estimate	Spatial cognition and LS					
Within-person bivariate change-to-change coupling effects (*δ*)	ΔSpatial Cognition_*T* – 1_ ➔ ΔLS*_T_*			ΔLS_*T* – 1_ ➔ ΔSpatial Cognition*_T_*		
	*β*	[95% CI]	*d*	*β*	[95% CI]	*d*
	−0.030	[−0.072, 0.013]	−0.085	−12.661*	[−22.892, −2.430]	−0.150
Within-person proportional change (*β*)	ΔSpatial Cognition_*T* – 1_ ➔ ΔSpatial Cognition*_T_*			LS_*t* – 1_ ➔ ΔLS*_t_*		
	0.171	[−0.129, 0.470]	0.069	−0.241	[−0.486, 0.005]	−0.120
	Spatial Cognition			LS		
Initial status						
Mean	36.655^***^	[35.571, 37.739]	4.111	2.556^***^	[2.491, 2.621]	4.804
Variance	131.221^***^	[113.427, 149.015]	0.896	0.433^***^	[0.359, 0.507]	0.707
Constant change (*α*)						
Mean	−7.583	[−18.211, 3.045]	−0.087	0.645*	[0.045, 1.245]	0.131
Variance	6.754	[−2.924, 16.431]	0.085	0.023	[−0.000, 0.047]	0.119
Residual error						
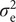	28.987^***^	[25.965, 32.008]	1.166	0.148^***^	[0.130, 0.165]	1.020
Model fit						
χ^2^	118.780					
*df*	40					
*p*	<.001					
CFI	.968					
RMSEA	.063					

*Abbreviations:* CFI, confirmatory fit index; CI, confidence interval; *df*, degrees of freedom; LS, life satisfaction; RMSEA, root-mean-square error of approximation; Δ, change in a parameter estimate at one time lag.

* *p* < 0.05.

^***^ *p* < 0.001.

#### Life satisfaction and verbal WM

Table 4 demonstrates that the BLCS model testing within-person change-to-future relations between LS and verbal WM had good model fit (χ^2^(*df* = 40) = 87.780, *p* < 0.001, CFI = .962, RMSEA = .052). Within-person decrease in LS at a 3–10-year time lag was significantly related to decrement in verbal WM during the adjacent subsequent time lag (*β* = 1.637, 95% CI [0.680, 2.594], *p* = 0.001, *d* = 0.208). Likewise, within-person decline in verbal WM at a 3–10-year time lag was significantly associated with reduction in LS at the next successive time lag (*β* = 0.357, 95% CI [0.051, 0.664], *p* = 0.022, *d* = 0.142). In addition, the change-to-future change relation for LS predicting verbal WM was significantly stronger than the reverse (Δχ^2^(*df* = 1) = 4.153, *p* = 0.042). Furthermore, time-lag length differences (3 vs. 11 year) did not significantly moderate the change-to-future change cross-construct coupling effects between verbal WM and LS (∆*χ*^2^(*df* = 2) = 2.195, *p* = 0.334).

**Table 4.** Bivariate latent difference score models of verbal WM and LS.

Parameter estimate	Verbal WM and LS					
Within-person bivariate change-to-change coupling effects (*δ*)	ΔVWM_*t* – 1_ ➔ ΔLS*_t_*			ΔLS_*t* – 1_ ➔ ΔVWM*_t_*		
	*β*	[95% CI]	*d*	*β*	[95% CI]	*d*
	0.357^***^	[0.051, 0.664]	0.142	1.637^**^	[0.680, 2.594]	0.208
Within-person proportional change (*β*)	VWM_*t* – 1_ ➔ ΔVWM*_t_*			LS_*t* – 1_ ➔ ΔLS*_t_*		
	0.478	[−0.026, 0.982]	0.115	−0.226	[−0.424, −0.027]	−0.139
	Verbal WM			LS		
Initial status						
Mean	4.749^***^	[4.631, 4.866]	4.909	2.561^***^	[2.496, 2.625]	4.813
Variance	1.007^***^	[0.784, 1.229]	0.553	0.433^***^	[0.360, 0.507]	0.707
Constant change (*α*)						
Mean	0.340	[−0.203, 0.884]	−0.110	0.612*	[0.104, 1.120]	0.147
Variance	0.077	[−0.006, 0.159]	0.076	0.027^**^	[0.008, 0.047]	0.167
Residual error						
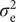	0.942^***^	[0.822, 1.063]	0.958	0.146^***^	[0.128, 0.164]	1.006
Model fit						
χ^2^	87.780					
*df*	40					
*p*	<.001					
CFI	.962					
RMSEA	.052					

*Abbreviations:* CFI, confirmatory fit index; CI, confidence interval; *df*, degrees of freedom; LS, life satisfaction; RMSEA, root-mean-square error of approximation; VWM, verbal working memory; Δ, change in a parameter estimate at one time lag.

* *p* < 0.05.

^**^ *p* < 0.01.

^***^ *p* < 0.001.

#### Life satisfaction and processing speed

Table 5 conveys that the BLCS model determining within-person change-to-future change associations between LS and processing speed had good model fit (χ^2^(*df* = 40) = 147.622, *p* < 0.001, CFI = .948, RMSEA = .072). Within persons, decline in LS at a prior 3–10-year time lag was significantly related to reduction in processing speed at the next time lag (*β* = 11.437, 95% CI [6.261, 16.613], *p* < 0.001, *d* = 0.354), and vice versa (*β* = 0.040, 95% CI [0.026, 0.054], *p* < 0.001, *d* = 0.269). Furthermore, the magnitude of change-to-future change relations was significantly stronger for LS predicting processing speed than conversely (Δχ^2^(*df* = 1) = 4.359, *p* = 0.037). In addition, time-lag length differences (3 vs. 11 year) did not significantly moderate the change-to-future change cross-construct coupling effects between processing speed and LS (∆*χ*^2^(*df* = 2) = 1.409, *p* = 0.494).

**Table 5.** Bivariate latent difference score models of processing speed and LS.

Parameter estimate	Processing speed and LS					
Within-person bivariate change-to-change coupling effects (*δ*)	ΔProcessing Speed_*t* – 1_ ➔ ΔLS*_t_*			ΔLS_*t* – 1_ ➔ ΔProcessing Speed*_t_*		
	*β*	[95% CI]	*d*	*β*	[95% CI]	*d*
	0.040^***^	[0.026, 0.054]	0.354	11.437^***^	[6.261, 16.613]	0.269
Within-person proportional change (*β*)	Processing Speed_*t* – 1_ ➔ ΔProcessing Speed*_t_*			LS_*t* – 1_ ➔ ΔLS*_t_*		
	0.105	[−0.046, 0.256]	0.085	−0.259^**^	[−0.431, −0.088]	−0.183
	Processing Speed			LS		
Initial status						
Mean	−41.698^***^	[−42.677, −40.720]	−5.182	2.597^***^	[2.531, 2.662]	4.881
Variance	112.772^***^	[96.305, 129.238]	0.833	0.417^***^	[0.344, 0.489]	0.699
Constant change (*α*)						
Mean	6.782*	[0.918, 12.646]	0.141	0.649^**^	[0.204, 1.094]	0.177
Variance	5.657^**^	[1.837, 9.476]	0.180	0.029^**^	[0.011, 0.047]	0.200
Residual error						
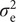	26.622^***^	[22.648, 30.597]	0.814	0.149^***^	[0.131, 0.168]	1.027
Model fit						
χ^2^	147.622					
*df*	40					
*p*	<.001					
CFI	.948					
RMSEA	.072					

*Abbreviations:* CFI, confirmatory fit index; CI, confidence interval; *df*, degrees of freedom; LS, life satisfaction; RMSEA, root-mean-square error of approximation; Δ, change in a parameter estimate at one time lag.

* *p* < 0.05.

^***^ *p* < 0.001.

## Discussion

Consistent with the foregoing theories, findings showed that across five time points over 23 years, larger reduction in LS at a time lag was notably related to greater decline in spatial cognition, verbal WM, and processing speed at a future successive time lag. Simultaneously, partially supporting Hypotheses 1 and 2, a larger decrease in LS at a time lag was preceded and associated with greater diminished verbal WM and processing speed, but not spatial cognition. Small yet significant within-person, cross-lagged, change-to-future change relations emerged across time (|*d*| = 0.142–0.354). These *within-person* change-to-future change relations held over and above self-feedback loops, regression to the mean, between-person effects, and prior scores. We offer some theoretical accounts for this pattern of findings to contribute to the literature on aging, cognitive functioning, and subjective well-being.

Why did a reduction in LS at a time lag consistently forecast a worsening of verbal WM, spatial cognition, and processing speed at the next time lag? This finding may be accounted for by scar theories, which propose and show that reduced LS and related mental health issues (e.g., excessive worry) can, over time, lead to decreases in cognitive functioning via heightened allostatic load [21,97]. This cascade of events may occur in the long term (e.g., 9–23 years) [24,25], plausibly via higher levels of markers of inflammatory activity (e.g., peripheral cytokines) and chronic stress (e.g., cortisol) in the bloodstream [23,98]. The continuing effect of decreased LS on allostatic load may alter cardiorespiratory and neurophysiological pathways in the amygdala and left precuneus, ventromedial prefrontal cortex, and striatum brain regions over long durations [99,100]. Importantly, these areas have been identified as essential for executive functioning, optimal reward processing, and emotion modulation [101]. Future longitudinal basic science studies can evaluate the strength of evidence for these propositions.

Simultaneously, what factors may account for decreases in processing speed and verbal WM preceding and predicting a decline in LS? Findings are concordant with theorists who propose the importance of processing speed [102,103] and WM [104] resources to fulfill goals and tasks to create a sense of gratification across development (*satisfaction-of-goals theory*) [49]. It is tenable that throughout life, these cognitive functioning domains promote higher levels of perceived control goal planning, execution, attainment, and related factors. Supporting these assertions, prior BLCS research showed that a decline in processing speed or WM coincided with the ability to perform activities of daily living [105], as well as a rise in trait negative affect and depression severity [7]. Subsequent cross-panel LCS mediation investigations with four or more assessment waves can test our proposed ideas.

Unexpectedly, change in spatial cognition was not related to future change in LS. This null finding might be due to the fact that the ability to mentally process and alter visual objects in real time as indexed by the DB and CR spatial cognition measures might not be as essential as WM and processing speed in everyday life. This conjecture awaits further empirical testing. In addition, the nonsignificant within-person change-to-future change in spatial cognition–LS connections herein were inconsistent with a prior OLS regression-based study of older adults [44], showing that between persons, higher spatial cognition predicted greater future LS. Differences in analytic data techniques (e.g., findings at the within-person level may not translate to the between-person level) [106], measures used, and sample attributes may contribute to any observed discrepancies in the literature. In addition, the effect of reduction in LS on future decline in spatial cognition, verbal WM, and processing speed was more substantial than in the opposite direction. Such findings might be attributed to how measures of cognitive functioning used herein might not necessarily reflect tasks of everyday living (e.g., house chores, grocery shopping, and balancing a checkbook) in community adults and, therefore, might not be as noticeable or bothersome [107]. These results could also be explained by how LS markedly affects daily motivation, mood, work and social functioning, and health behaviors [108,109], factors that likely impacted various cognitive domains. Future empirical research work can test these possibilities.

The current study has some limitations. One limitation was the use of two spatial cognition tests, one processing speed test, and one WM test. Future research could clarify if other important cognitive function domains (e.g., verbal fluency) [7,110]) would be related to LS with comprehensive multiple-item cognitive tests. Subsequent studies should also include subjective cognitive function measures since they may differ from performance-based ones and function as early warning signs of incident dementia [111]. Furthermore, our study included one time lag that was significantly longer than the others, which may have influenced our findings. However, we determined that the pattern of results did not differ substantially based on the time-lag length, and we used the least biased method of analyzing data with different lag lengths [91]. Another possibility is that systematic attrition impacted our findings. Nonetheless, we used a gold standard data replacement strategy that accounts for systematic associations between study variables and missingness. In addition, the oral SDM test used herein tends to yield higher scores than written graphomotor forms [71]. However, given that the current study focused entirely on change in the same test over time, higher scores would likely be present at all time points and would not have affected our current findings. Future studies should use processing speed tests that comprise both oral and written components to obtain more reliable estimates of individuals’ processing speed abilities. In addition, the tests used herein had poor ecological validity because these tasks (e.g., BD) are rarely if at all, required in day-to-day living in the real world, thus highlighting the importance of using ecologically valid cognitive functioning measures (e.g., [112]) in future studies. Relatedly, unmeasured third variables (e.g., motivation, self-efficacy, perceived social connectedness, and positive affect) [113,114] should be considered. Finally, cognitive functioning tests, in particular, can be associated with practice effects, particularly across relatively shorter retest intervals of less than 2 years [115–117]. However, we do not believe that practice effects explain our significant results for several reasons. First, our time lag length was 3–11 years, and at least one study found no cognitive improvement across three years [116]. Second, our analyses examined the bidirectional relationship between waning LS and *worsening cognitive functioning.* If there were any impact of practice effects on cognitive functioning, this would mask (rather than enhance) cognitive functioning decline and likely diminish the bidirectional within-person association between reduced LS and worsening cognitive function. These shortcomings aside, study strengths include the well-powered sample size, study novelty, and use of multiple assessments to capture cognitive function that minimized measurement error. Furthermore, the current study utilized a suitable advanced SEM method that permits inferences of within-person change-to-future change relations to evaluate the preceding theories and advance literature empirically.

If the pattern of results herein is reproduced, the current study has some clinical implications. First, meditation- and mindfulness-based interventions have increasingly shown promise to enhance sustained attention, WM, spatial attention, and related cognitive function markers [118–120], or protect against their worsening decline across long durations in highly stressful situations [121,122]. In addition, lifestyle-based (e.g., cognitive-behavioral therapies, dancing, and yoga) [123,124] and cognitive function interventions (e.g., executive functioning or memory training, and reminiscence therapy) [125–128] have been shown to boost cognitive function or LS in adults. However, the state of research on this topic is nascent. Thus, conducting more gold standard randomized controlled trials can aid in drawing more definitive conclusions on their efficacy to enhance LS and cognitive function at the prevention and treatment stages across adulthood. In addition, based on emerging evidence [129], clinical science can profit from future studies by testing if cognitive function serves as a treatment predictor, moderator, or mediator, to better understand mechanisms of change and optimize treatments.

## Data Availability

The Swedish Adoption/Twin Study on Aging (SATSA) publicly available dataset used herein was obtained via the Inter-University Consortium of Political and Social Research (ICPSR) data repository website (https://www.icpsr.umich.edu/icpsrweb/NACDA/studies/3843).

## Footnotes

1 Participants diagnosed with dementia using the Diagnostic and Statistical Manual of Mental Disorders-Third Edition [60, 61] were removed to understand the change-to-future change relations between life satisfaction and unique cognitive functioning domains in initially cognitively intact community adults. Nonetheless, a similar pattern of findings emerged when these participants were included.

2 See [71] for updated norms of this test.

3 Results of the analyses were similar with the unimputed dataset.

## Supplementary material

For supplementary material accompanying this paper visit https://doi.org/10.1192/j.eurpsy.2022.19. click here to view supplementary material
