# Supplementary material for: Life satisfaction prevents decline in working memory, spatial cognition, and processing speed: Latent change score analyses across 23 years
Source: Eur Psychiatry. 2022 Apr 19;65(1):e27. doi: 10.1192/j.eurpsy.2022.19 (PMC9121850; doi:10.1192/j.eurpsy.2022.19)
Supplement: Supplementary file 1 [file S0924933822000190sup001.docx]

Table S1

*Demographics Table*

|  | *M* (or *N*) | *SD* (or %) | |
| --- | --- | --- | --- |
| Female (%) | 302 | (58.10) | |
| Formal education (%) |  |  | |
| College/Higher education | 366 | (70.40) | |
| High school | 110 | (21.20) | |
| Lower secondary or elementary school | 44 | (8.46) | |
| Age (*M* (*SD*)) |  |  | |
| Wave 1 | 59.61 | (8.98) | |
| Wave 2 | 62.63 | (8.97) | |
| Wave 3 | 65.63 | (8.98) | |
| Wave 5 | 75.55 | (8.15) | |
| Wave 6 | 77.42 | (7.39) | |
|  |  | |  |

Table S2

*Items in the Life Satisfaction Measure*

| **Item No.** | **Question** | **Response Format** |
| --- | --- | --- |
| 1 | As I get older everything seems to be better than expected. | 1 = *Strongly disagree* to 5 = *Strongly agree* |
| 2 | I have had more luck in my life than most people I know. |  |
| 4 | I am as happy now as I was when I was younger. |  |
| 5 | These are the best years of my life. |  |
| 7 | I am just as interested in what I’m doing nowadays as I was in the past. |  |
| 8 | I can look back on my life with satisfaction. |  |
| 9 | I have planned what to do in the near future. |  |
| 12 | Most of my expectations in life have been fulfilled. |  |
|  |  |  |

*Note.* These 8 items were positively-keyed life satisfaction items. The following negatively-keyed items from the original scale (Harris, Pedersen, Stacey, McClearn, & Nesselroade, 1992) were removed (number in parentheses denote item no. in the original scale): (3) This is the most boring period in my life. (6) Nearly everything I do is boring or monotonous. (10) Looking back on my life, I realize that my greatest expectations have not been fulfilled. (13) I get depressed or low more often than other people. Although prior studies have used the 13-item life satisfaction scale (e.g., Harris et al., 1992), we chose to use an 8-item version that included only the positively-keyed items. This is because research has found consistently that respondents tend to misconstrue negatively-keyed items to the point that changing responses from being negatively- to positively-keyed does not account for construal-related variability (Salazar, 2015). Incorrect construal of negatively-keyed items reduces the internal consistency of scores and misrepresents the factor structure (e.g., revealing a two-factor structure solely based on phrasing valence when the construct is in fact unidimensional) (Irions, 2018; van Sonderen, Sanderman, & Coyne, 2013). Moreover, respondents might unintentionally highly endorse negatively-keyed items when they meant to state the opposite (van Sonderen et al., 2013). Moreover, as shown in the table below, a series of confirmatory factor analyses showed that the 8-item life satisfaction scale had significantly better model fit at all time-points.

|  | 13-item LS | | |  | 8-item LS | | |
| --- | --- | --- | --- | --- | --- | --- | --- |
|  | χ^2^(*df*) | CFI | RMSEA |  | χ^2^(*df*) | CFI | RMSEA |
| Time 1 (1987) | 191.585^***^ (65) | .947 | .061 |  | 58.980^***^ (20) | .968 | .061 |
| Time 2 (1990) | 171.708^***^ (65) | .949 | .056 |  | 40.251^***^ (20) | .980 | .044 |
| Time 3 (1993) | 180.872^***^ (65) | .937 | .059 |  | 35.207^*^ (20) | .987 | .038 |
| Time 4 (2004) | 212.828^***^ (65) | .927 | .066 |  | 53.918^***^ (20) | .973 | .057 |
| Time 5 (2007) | 260.703^***^ (65) | .913 | .076 |  | 79.432^***^ (20) | .946 | .076 |
|  | Comparison between 13-item and 8-item LS | | |  |  |  |  |
| Change in fit indices | ∆χ^2^(*df*) | ∆CFI | ∆  RMSEA |  |  |  |  |
| Time 1 (1987) | 132.604^***^ (45) | -.022 | .000 |  |  |  |  |
| Time 2 (1990) | 131.458^***^ (45) | -.032 | .012 |  |  |  |  |
| Time 3 (1993) | 145.664^***^ (45) | -.050 | .020 |  |  |  |  |
| Time 4 (2004) | 158.910^***^ (45) | -.046 | .009 |  |  |  |  |
| Time 5 (2007) | 181.272^***^ (45) | -.033 | .004 |  |  |  |  |
|  |  |  |  |  |  |  |  |

*Note.* CFI = confirmatory factor analysis; RMSEA = root mean square error of approximation; *df* = degrees of freedom; LS = life satisfaction; ∆ = change in fit indices.

Table S3

*Descriptive Statistics and Between-Person Correlation Matrix of Key Study Variables*

|  | 1 | 2 | 3 | 4 | 5 | 6 | 7 | 8 | 9 | 10 |
| --- | --- | --- | --- | --- | --- | --- | --- | --- | --- | --- |
| 1. T1LS | – |  |  |  |  |  |  |  |  |  |
| 2. T2LS | .592^***^ | – |  |  |  |  |  |  |  |  |
| 3. T3LS | .520^***^ | .603^***^ | – |  |  |  |  |  |  |  |
| 4. T4LS | .174^***^ | .156^***^ | .201^***^ | – |  |  |  |  |  |  |
| 5. T5LS | .192^***^ | .198^***^ | .224^***^ | .214^***^ | – |  |  |  |  |  |
| 6. T1GC | -.001 | .016 | .038 | .019 | -.032 | – |  |  |  |  |
| 7. T2GC | -.023 | .010 | .029 | -.014 | -.100^*^ | .640^***^ | – |  |  |  |
| 8. T3GC | -.028 | -.007 | .032 | .016 | -.115^**^ | .671^***^ | .813^***^ | – |  |  |
| 9. T4GC | .002 | .015 | -.049 | -.013 | -.135^**^ | .405^***^ | .550^***^ | .559^***^ | – |  |
| 10. T5GC | -.041 | -.039 | -.099* | -.052 | -.017 | .325^***^ | .397^***^ | .425^***^ | .419^***^ | 1 |
| Mean | 2.641 | 2.693 | 2.735 | 2.797 | 2.798 | 0.084 | -0.045 | -0.125 | -0.158 | -0.004 |
| *SD* | 0.724 | 0.593 | 0.564 | 0.589 | 0.563 | 3.134 | 3.131 | 3.258 | 3.175 | 3.159 |
| Skewness | 0.566 | 0.312 | 0.337 | 0.360 | 0.212 | -0.093 | -0.042 | 0.001 | 0.001 | -0.126 |
| Kurtosis | 0.284 | 0.368 | 0.243 | 0.101 | 0.082 | -0.227 | -0.279 | -0.330 | -0.483 | -0.405 |
| Minimum | 1.000 | 1.250 | 1.375 | 1.125 | 1.000 | -9.290 | -9.101 | -9.916 | -8.644 | -8.535 |
| Maximum | 5.000 | 4.625 | 4.625 | 4.625 | 4.750 | 8.388 | 7.774 | 8.958 | 7.341 | 7.242 |

*Note.* ^*^*p* < .05; ^**^*p* < .01; ^***^*p* < .001. *SD* = standard deviation; LS = life satisfaction; GC = global cognition; T1 = Time 1 (1987); T2 = Time 2 (1990); T3 = Time 3 (1993); T4 = Time 4 (2004); T5 = Time 5 (2007).

Figure 1

*Attrition Across All Five Waves of Assessments*

*Note.* LS = life satisfaction.
